# A Standardised Abundance Index from Commercial Spotting Data of Southern Bluefin Tuna (*Thunnus maccoyii*): Random Effects to the Rescue

**DOI:** 10.1371/journal.pone.0116245

**Published:** 2014-12-26

**Authors:** Marinelle Basson, Jessica H. Farley

**Affiliations:** CSIRO Oceans and Atmosphere Flagship, Hobart, Tasmania, Australia; Aristotle University of Thessaloniki, Greece

## Abstract

Commercial aerial spotting of surface schools of juvenile southern bluefin tuna (SBT), *Thunnus maccoyii*, is conducted as part of fishing operations in the Great Australian Bight in summer. This provides the opportunity to efficiently collect large amounts of data on sightings of SBT. The data can potentially be used to construct a time-series index of relative abundance by standardising the data for issues such as weather, spotter ability and ocean conditions. Unlike a statistically designed survey, the commercial spotting is governed by business considerations and fishing operations. The SBT dataset is therefore highly unbalanced with regard to spotters operating in each season. This complicates the standardisation of the data, particularly with regard to interactions between covariates. We show how a generalized additive model with random effects can simplify both the fitting of the model and the construction of an index, while also avoiding the need to leave out strata or interaction terms that are important. The approach is applicable to standardisation of more traditional catch and effort data.

## Introduction

Fish stock assessments rely on estimates of and trends in population abundance. This paper presents a method for modelling data from aerial sightings of fish, or schools of fish, made as part of commercial fishing operations to construct a standardised index of relative abundance. In particular, we show how a highly unbalanced dataset can be modelled by treating some factors and interactions between covariates as random effects. This avoids the need to remove some ‘strata’ (e.g. months or spotters) from the dataset, and appropriately deals with strata which have few observations.

Our example dataset is derived from commercial aerial observations of juvenile southern bluefin tuna (SBT) *Thunnus maccoyii*. During summer in the Great Australian Bight (GAB), SBT form surface schools of similar-sized fish, which produce a distinctive ripple on the water's surface when feeding or swimming [1]. These surface schools can be seen from the air and spotter aircraft are used to assist in locating schools so they can be captured by purse seine vessels for grow-out in sea cages (see e.g. [2]).

Commercial SBT spotting data collected in the 1980s and 1990s had previously been analysed and found to be problematic [3]. This type of data can suffer from exactly the same shortcomings that fisheries catch and effort data suffer from (e.g. operational changes, gear/equipment improvements that are hard to quantify, inconsistent or incomplete recording of data). Quantifying “effort” for commercial spotting flights can be difficult, particularly if flight paths and activities are only recorded on coarse temporal and spatial scales. Because of these issues with commercial spotting data, a scientific line-transect aerial survey with consistent design and protocols was developed for the GAB. It was established in 1993 to form the basis of a fishery independent index of relative abundance for ages 2–4 years [4]. This survey has been conducted annually from 1993 to 2000 and from 2005 to 2013, and encompasses a large area of the GAB from 128°E to 135°E, from the coast to just over the continental shelf break. The suspension of the survey during 2001 was due to logistical problems, but it was reinstated in 2005 [4], [5], and is now a key index of juvenile abundance in the assessment and management procedure for SBT [6].

During the suspension of the aerial survey the fishing industry offered to collect commercial spotting data and to make those data available for analyses. The relevance of these data is clear considering the high value of the fishery, and the status of the SBT stock at the time. In 2001 the SBT spawning stock biomass (SSB) was estimated to be well below the unfished level; depending on the assessment method used, estimates ranged from 13–19% to as low as 4–11% [7]. Recruitments in the 1990s were estimated to be less than half those in earlier years [7], and continued direct monitoring of recruitment was considered to be important. In 2002 the CSIRO therefore started a program of collecting commercial spotting data annually and developing a standardised index of relative juvenile abundance. The program of collecting and analysing commercial spotting data has continued, and it provides an additional indicator of trends in juvenile abundance.

Standardisation is necessary to obtain a consistent – comparable from year to year – index of abundance. There are at least three aspects to consider: weather conditions, the ability of each spotter, and fish behaviour. Factors such as cloud cover, sea state, wind and visibility will affect any spotter's ability to see surface schools of fish. Even under identical conditions, spotters differ in their ability to see schools of fish and provide estimates of the school size. Fish surfacing behaviour may differ depending on oceanographic conditions, and if there are trends in these conditions it would be particularly important to identify by inclusion in a standardisation. It is beyond the scope of this paper to fully explore and identify the environmental drivers of surfacing and schooling behaviour (but see [8], [9] for research in this field).

The standardisation of traditional catch-effort data is familiar enough [10], [11], but there are few examples of attempts to standardise fish spotter data. Squire [12] considers sightings per unit effort of six pelagic fish species off California and Baja California, but made no attempt to standardise for differences in spotting conditions or between spotters. Lo et al. [13] analyse fish spotter data from the northern anchovy fishery off California using delta-lognormal models to derive standardised indices. Lutcavage et al. [14] and Lutcavage and Kraus [15] attempted to use photographic and commercial spotting data for giant bluefin tuna (*Thunnus thynnus*) in New England waters as a basis for an index of abundance. However, they comment that the ultimate aim is to conduct a fishery independent aerial survey.

Using our SBT dataset as an example, we show how commercial spotting data can be analysed in a generalized additive mixture model (GAMM, [16]) framework, and how a standardised index of relative abundance can be constructed. Although an agreed set of protocols are used to record and collect the data (see Methods) in order to minimise inconsistencies, there is no control over how many and which spotters operate in any season. For example, one spotter has operated in all fishing seasons, another only in two. This implies a highly unbalanced dataset with regard to spotter and is the main reason for using random effects rather than a more conventional generalized linear or additive modelling framework (GLM or GAM). The GAMM framework can also be used to standardise with the more familiar commercial catch and effort data from trawl fisheries, particularly if important strata have few observations.

## Methods

### Search effort and SBT sightings data

Commercial aerial spotting data were collected voluntarily by experienced commercial tuna spotters searching for SBT in the GAB during the main purse seine fishing season, from December 1 to March 31 (note ‘fishing season’ refers to the year associated with March). Data were collected for the 2002 to 2013 fishing seasons but not all relevant covariates were recorded in the first season, so we use data for seasons 2003 to 2013.

The spotting data were collected using the same set of protocols in all seasons. Each plane had both a spotter and a pilot. For most flights, the spotter searched the sea surface on both sides of the plane for surface schools of SBT. During some flights, the pilot may also have searched for schools and these data were included in our analyses. A GPS with track plotting and waypoint recording facilities was used to record the flight path of the aircraft each day, which was downloaded to a laptop computer. The flight path is a series of latitudes, longitudes and times recorded every 10 to15 seconds. A GPS waypoint (position and time) was recorded at the start and end of “search effort” during each flight. When a “sighting” of SBT was made, a waypoint was recorded over the school, or schools. Most SBT sightings are recorded as single schools (∼80–90% by season), but some are recorded in groups of 2–10 or even 50+ schools. The spotter estimated a range for the size of fish in each school (in kg) and the biomass of each school (in tonnes) in a logbook. On the rare occasion when a school with a mix of species was encountered, the spotter only recorded the fish size and biomass of the SBT component of that school. Only the information on biomass of each school is used in our analysis. Since SBT are highly mobile, it is possible for spotters to record the same school at different locations on a single day. However, spotters argued that this rarely occurred, and these schools were identified (i.e., indicated by spotters) in the logbooks and not included our analyses. Also, schools may well be spotted and counted on different days; this cannot be avoided. However, since we only develop a *relative* index of abundance, it should not be sensitive to double-counting provided there is no systematic trend in the extent of double-counting. GPS flight path data were not available for 6% of flights due to logistical issues. However, the position and time for search effort (start/stop) and the locations of SBT schools were still recorded.

The purse seine fishery also potentially targets other species, such as skipjack tuna (*Katsuwonus pelamis*) or blue mackerel (*Scomber Australasicus*), in which case the spotters may not just be searching for SBT. The target species of each flight (SBT, skipjack tuna, mackerel, or a combination of these) was also recorded since the 2003 season.

The duration of “search” sectors during flights were calculated using the logged position and time. The logbook data on SBT sightings were summarised to give the total number of sightings, schools, and total biomass per spotter per day. Flights were excluded if they were less than 30 minutes duration because these were considered too short to have meaningful search effort. As these data were removed for all seasons, it should not affect the relative index of abundance. The data were compiled as a set for the entire area and all the analyses were done on the ‘whole area’ dataset.

### Environmental variables

Environmental observations were recorded at the start of each spotting flight and when the conditions changed significantly during the day. The environmental observations included wind speed (knots) and direction (8 compass directions), air temperature (°C), visibility (distance that SBT schools can be observed; nautical miles), and categorical quantities for cloud cover (0–8), spotting conditions (rating by the spotter on general conditions; 0–5 poor to good) and swell height (0–3). The environmental variables are calculated as the means for that day and spotter.

Moon phase was obtained for each spotting day because it is thought to influence SBT behaviour, and it has been a significant factor in the “sightings per mile” component of the analysis of the line-transect aerial survey data [17].

### Modelling approach

The intention of modelling is to estimate a standardised relative index of juvenile abundance. The model must be able to cope with several data issues: days where no SBT were observed (i.e. zeros in the data), a strong dependency of the variance on the mean, and, most importantly, a highly unbalanced dataset. Fishing and spotting operations usually start in December and last until March, but in the 2010 and 2013 seasons, they ended in February, so there are no data for March. Missing or limited data for spotters is a more serious problem. The number of spotters operating in any given season has varied between 2 and 6 (Table 1). Up to 2012 only two spotters operated in all the seasons and it was possible, though not ideal, to use only their data in a generalized linear model (GLM). In 2013, however, only one of these two spotters operated (together with two others who had operated in the past). This was a strong incentive for developing a modelling approach that could handle the unbalanced dataset and interactions between covariates with missing data in some strata.

**Table 1:** Search effort and SBT sighted by commercial spotters in the 2002–2012 fishing seasons.

Fishing season	No. Spotters	No. Flights	Search effort (hrs)	% flights with SBT recorded	Total number of schools	Total biomass* recorded	% of effort by spotters 1 and 6
2003	6	102	425	82.4	1301	38559	49.7
2004	4	118	521	77.1	1133	33982	65.4
2005	5	116	551	94.0	2395	87447	66.2
2006	4	102	452	82.4	1554	50524	73.7
2007	4	120	600	91.7	2600	94018	66.8
2008	3	93	451	80.6	2529	100341	76.3
2009	4	114	527	77.2	1353	41514	80.4
2010	4	49	210	83.7	918	32907	79.6
2011	2	64	328	95.3	1472	75887	100.0
2012	2	73	378	87.7	799	31959	100.0
2013	2	77	362	83.1	1529	67911	55.3

*The total biomass recorded does not represent the total absolute biomass of SBT present in the survey area, as many schools were potentially recorded several times (either by different spotters on the same day or over several days).

We chose a GAMM approach with spotter and 2-way interaction terms (between spotter and season, and between season and month) treated as random effects. Dealing with zero observations and the mean-variance relationship is addressed by using the Tweedie family of distributions [18], [19] with a log-link, so that different factors combine multiplicatively. The mean-variance relationship in Tweedie distributions follows a power-law with adjustable exponent Φ, with 1<Φ<2. The value of Φ = 1 coincides with the Poisson distribution, and a value of Φ = 2 with the Gamma distribution. For Φ<2 zero observations cause no problems. The exponent is not directly estimated, but a range of values are explored and checked for appropriateness by graphical means; a smooth fitted through the residuals should be linear without trend [16].

It is likely that sightings would have non-linear relationships with some of the environmental covariates. For example, SBT have been shown to occur within a preferred range of temperatures [20], which may imply a parabolic relationship between sightings and temperature; this is accommodated in the model by fitting curvilinear rather than linear relationships (i.e. using a GAM rather than a GLM framework [21])

Extensive preliminary analyses showed that cloud cover, swell, visibility and moon phase are not significant, and the resulting index is not sensitive to their inclusion or exclusion. The more parsimonious model, referred to as the final model, for the log of biomass (*b*) observed by a particular spotter on a given date is specified by the following formula in R [22]:
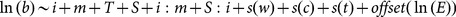
(1)where season (*i*), month (*m*) and target species (*T*) are factors fitted as fixed effects, spotter (*S*) and the two-way interactions between season and month (*i:m*) and spotter and season (*S:i*) are fitted as random effects. The other covariates (wind = *w*, spotting conditions = *c*, temperature = *t*) are fitted as smooth terms with thin plate regression splines as the basis and *k* the dimension of the basis. The choice of *k* is not critical, but this was checked by extracting the deviance residuals and refitting a model with just one of the covariates at a time [16]. The last term in the formula represents the sighting effort (*E*) associated with the observed biomass. For comparison, a simpler model without the two interaction terms was also fitted. An important advantage of using random effects (RE) is that when little or no data exist for a given level of a term (say for a particular spotter and month combination), we still have information about it because we are assuming it comes from a normal distribution with a certain mean and variance which are estimated within the model.

All analyses were done in R using the “gam” function from the library “mgcv” [21], using the “paraPen” option to fit the RE components of the model. The estimation method was restricted maximum likelihood (REML).

The Akaike Information Criterion (AIC) statistic was used to compare model fits following diagnostic checks to ensure adequate fit. We also evaluated the sensitivity of the standardised index to the choice of model and Tweedie parameter (Φ).

In the context of SBT, our goal is to construct an annual relative index of juvenile abundance that can be meaningfully compared across seasons. To do so, we need to estimate what the biomass of SBT spotted would have been in each season under standardised conditions (i.e., with the same spotter, environmental conditions, targeting behaviour, and search effort). The first step in constructing such an index is to set up a prediction dataset with each of the included environmental covariates and the ‘targeting’ covariate having the same value in each month and season. Any value can be used for the covariates since the index reflects relative rather than absolute abundance. We chose median values for the environmental covariates and “SBT” for the targeting covariate. The search effort (offset term) is set to 1. The prediction dataset is constructed for one spotter and for all months and seasons in order to include the season:month interaction term. This term is important because (we assume) that it reflects real differences in abundance. On the other hand, the spotter:season interaction is considered to be noise, i.e. variability in spotting ‘effectiveness’, and is therefore not included. The single spotter is also assumed to operate at the same level of effectiveness in each season. Predicted values are obtained by first generating the model matrix (using the ‘predict.gam’ function in R, with type = “lpmatrix”), setting to zero the columns coinciding with the spotter:season interaction terms and the spotters not being used for prediction and then multiplying by the vector of coefficients. The result is predicted biomass for each month (*m*) and season (*i*), say *p_m,i_*, on the log scale. The quantities of interest on the response scale are simply *x_m,i_*  =  exp(*p_m,i_*). These predicted biomass values are summed over months within each season to give a seasonal total, *U_i_*: 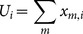
(2)


This index is used as an indicator of trends in juvenile abundance, and it is convenient to present the index scaled to its long term mean. The index *U_i,_* for season *i = 1,…n* is divided by the mean of the seasonal values:
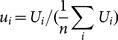
(3)


Since the mean itself has associated uncertainty, this needs to be propagated through to the covariance matrix of the standardised index. The chain rule is used to calculate a matrix of numerical derivatives for the functions applied to the ‘raw’ predictions with respect to the coefficients in the model. Let this matrix be *D*, then the covariance matrix, *v*, on the log scale is:
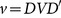
(4)where *V* is the covariance matrix of the model fit (an output of the “gam” function in R). Assuming log(*u_i_*) has a normal distribution, 95% confidence intervals are calculated using the delta method so that the lower (CI.025) and upper values (CI.975) are:



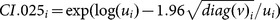
(5)

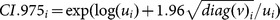



The line-transect aerial survey and commercial spotting indices overlap in the period 2005 to 2013. In order to directly compare them, the aerial survey index was scaled to the mean ( = 1.12) of the commercial spotting index over 2005 to 2013.

## Results

### Search effort and SBT sightings

SBT sightings data were collected during 1028 flights undertaken between 2003 and 2013 (Table 1). The number of spotters required by industry has decreased as there has been a tendency over time for fewer fishing companies to catch their own tuna, but rather relying on another company (and their spotter) to locate and catch their quota of SBT. Note that the total biomass shown in Table 1 does not represent the total biomass of SBT present in the survey area, as schools were potentially recorded several times (either by different spotters on the same day or over several days), and some schools may have been missed. For this reason it is only reasonable to estimate a relative index of abundance and not an absolute index of abundance.

**Figure 1:**
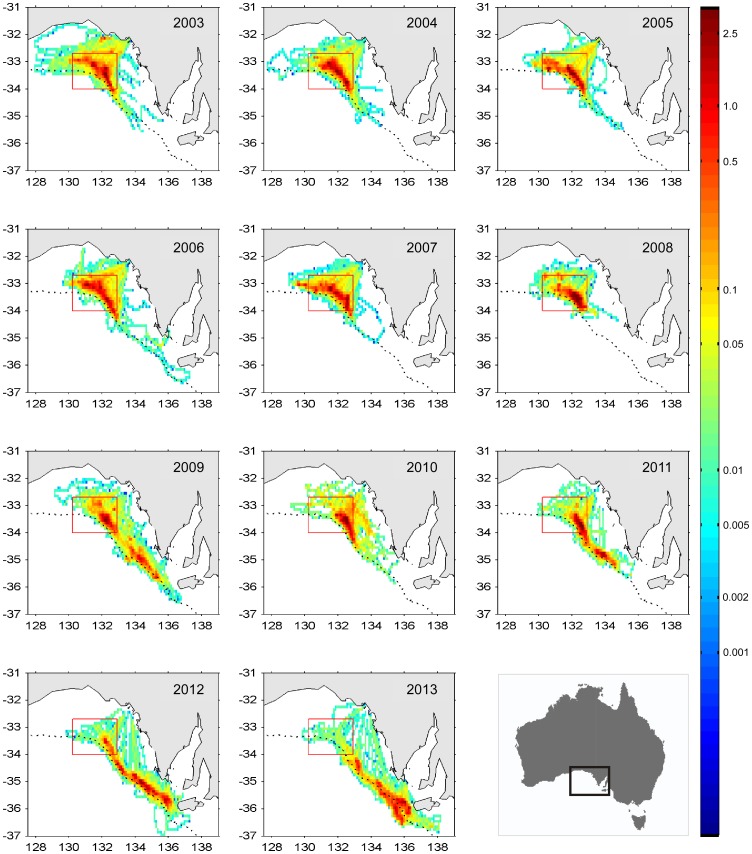
Location and intensity of search effort in the Great Australian Bight by fishing season. Search effort is measured in nautical miles flown per 0.1° square. The core fishing area is shown by a red square for reference, and the dotted line indicates the 200 m depth contour (shelf break). Search effort for flights where GPS tracks were not available is not included.

In 2003–2008 and 2010, the location of SBT sightings varied little (Fig. 1). The areas of highest SBT sighted per nautical mile searched occurred within the same ‘core fishing area’ (130–133°E and 33–34°S) which was commonly searched in previous years (see [3]). In 2009 and again in 2011–2013, a significant amount of search effort occurred to the southeast of the core area.

### Model results

**Figure 2:**
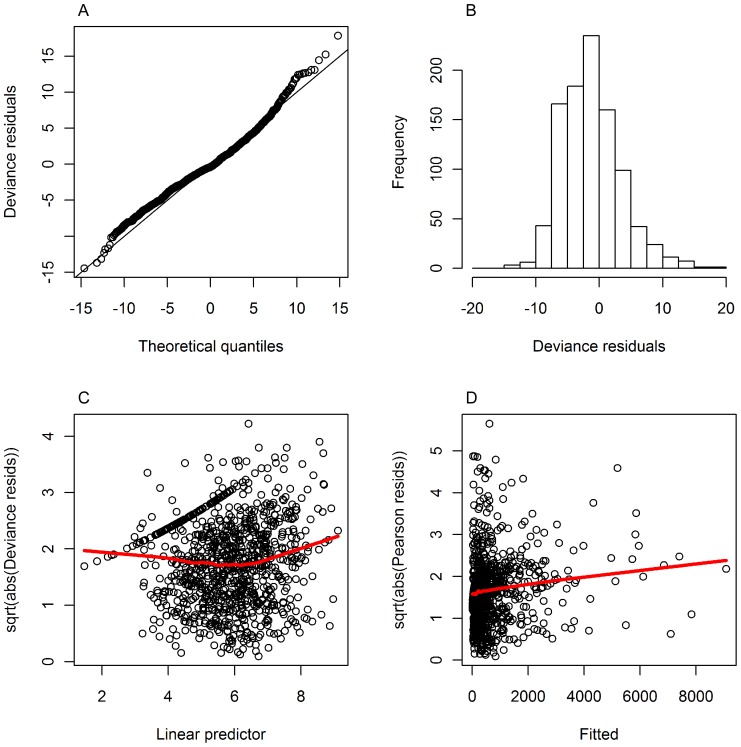
Standard diagnostic plots for the GAM defined in Eqn. 1. Plots produced by mgcv-package in R: A) qq-plot, B) histogram of the deviance residuals, C) square root of absolute deviance residuals against the linear predictor, D) square root of the absolute Pearson residuals. The solid red lines in panels C and D are smooths fitted to the residuals to help highlight any strong trends that may be present.

Diagnostics for the model (eqn. 1) show no trends or patterns in the residuals (Fig. 2). The qq-plot is more linear than for a model without the interaction terms, which has a fatter upper tail in the distribution of residuals. The model without interaction terms also has a larger AIC value (12789) compared to the model with interactions (12703).

The relationship between the square root of absolute deviance residuals and the linear predictor (solid red line, Fig. 2C) is sufficiently flat to indicate that the assumed Φ-value for the Tweedie distribution is acceptable. The value of Φ = 1.47 was obtained by visual inspection of this figure; smaller values of Φ lead to positive slope in the line; larger values of Φ lead to a more curved and/or negatively sloping line. The resulting index is not, however, sensitive to the choice of Φ.

We also looked at deviance residuals plotted against covariates included in the model and there were no remaining patterns. The target species covariate “mackerel” has poor residuals though this is not surprising given the small sample size of 15 observations (Table 2).

**Table 2:** Estimates of coefficients (on the response scale) for the targeting covariate treated as a factor relative to SBT.

Category	Coefficient	%CV	N
SBT	1	-	806
SBT and Skipjack	1.16	16	49
SBT and Mackerel	0.59	21	40
SBT and Skipjack and Mackerel	0.71	32	18
Skipjack	0.69	17	52
Mackerel	0.57	34	15
Skipjack and Mackerel	0.21	121	2

The percent coefficient of variation (CV) and number of observations (N) in each category are also given.

A check of residuals relative to covariates that are NOT included in the model also show no patterns. An additional check for the appropriateness of the effective degrees of freedom for the smoothed covariates was run by extracting the deviance residuals and refitting a model with just one of the covariates at a time. In all cases the resulting model had estimated degrees of freedom values close to zero.

Flights targeting both skipjack and SBT were slightly more effective than those just targeting SBT, though the sample size is substantially smaller – 49 versus 806 for SBT only (Table 2). Most other targeting categories were between 55–70% as effective, but ‘skipjack and mackerel’ flights were only 21% as effective (Table 2).

The estimated coefficients of variation (CVs) for the spotter random effects are between 37% and 46%, with those spotters that have fewer observations in the dataset having the higher CVs (Table 3). Results also confirm that, on average, the spotters differ in their ability to spot SBT but the extent of the difference between them varies by season. This is highlighted by the improvement in model fit when an interaction term between spotter and season is included, and the fact that the standard deviation of the random effects for this interaction term is not negligible (0.31). There are no trends in the season-specific coefficients (i.e. from the interaction term) for the spotters that have operated almost continuously (a, b and c; Table 3), and their coefficients do not track one another.

**Table 3:** Estimated coefficients of variation (CV) for Spotter treated as a random effect.

Spotter	Seasons	Days	%CV
a	11	362	37
b	10	330	38
c	8	101	39
d	6	77	39
e	3	83	41
f	2	29	46

The number of seasons each spotter has operated and the total number of days (i.e. records in the dataset) are also listed.

**Figure 3:**
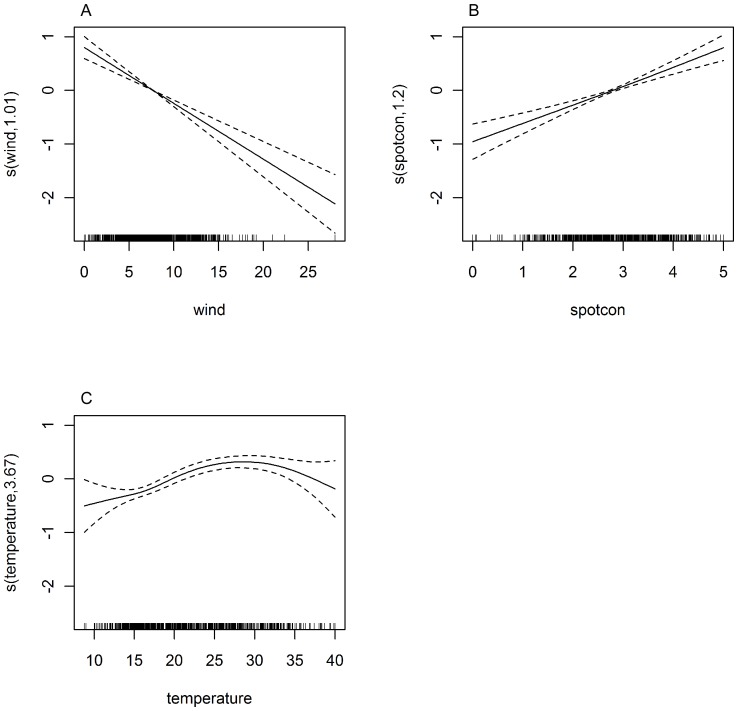
Estimated smooth relationships with 95% CI's between log(biomass spotted/search effort) and covariates. Vertical axis labels show covariate and effective degrees of freedom of the smooth (e.g. s(wind, 1.01) in panel A). A) ‘wind’ (windspeed in knots), B) ‘spotcon’ (spotting conditions between 0 and 5) and C) ‘temperature’ (mean air temperature in °C). The ‘rug’ on the horizontal axis shows where datapoints are located.

The estimated relationship between sightings and wind speed is negative, and between sightings and spotting conditions is positive (Fig. 3). There is a strong correlation (0.63) between spotting conditions and wind, but leaving one or the other out of the model does not improve the fit; neither does it affect the resulting abundance index. The relationship between sighting rate and temperature is dome-shaped with a peak at around 28°C (Fig. 3).

### Index of Abundance

**Figure 4:**
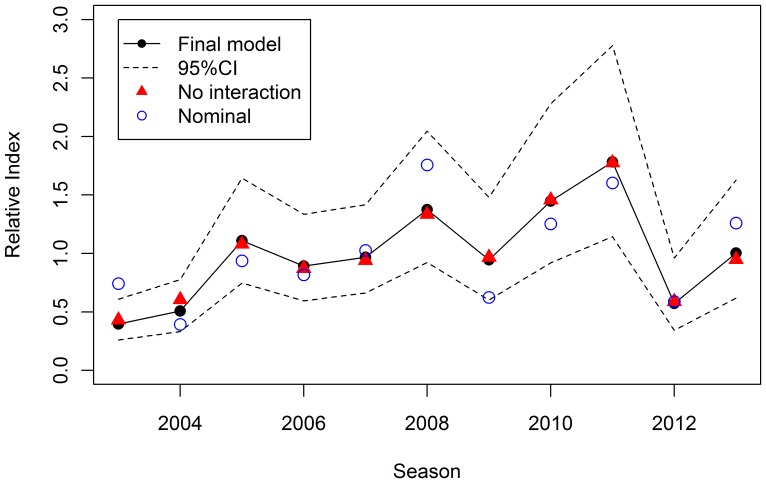
The standardised annual index of relative juvenile abundance from the final model with interactions. The index derived from the final model scaled to the mean with approximate 95% confidence interval. Also shown are index values (but not uncertainty) for a model with no interaction terms and the nominal (i.e. unstandardised) index.

The estimated annual index of relative juvenile abundance (Fig. 4) is not sensitive to model choice. There is only a 4.5% average difference between an index from the final model (with interaction terms) and an index from a model without interactions. The maximum difference is only 19% (for 2004) and the overall time-trends are almost identical (Fig. 4). The index is also not sensitive to the choice of Tweedie parameter within the range of values that provide acceptable fits.

The index value for 2008 appears to be most sensitive to the different model detail. This is also the season during which a large amount of biomass was observed, the nominal index is the highest in the series, but spotting conditions and visibility were particularly good. The standardisation therefore has a stronger effect than in the other seasons (Fig. 4).

**Figure 5:**
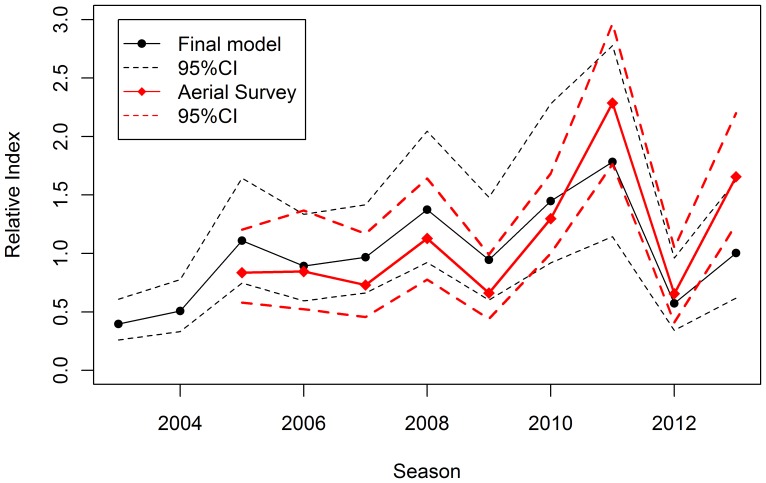
Comparison of standardised index and line-transect aerial survey index taken from Eveson et al. (2013). The standardised index from the final model as in Figure 4 compared to the line-transect aerial survey index (scaled to the commercial index mean over 2005–2013); 95% confidence interval s for each index are also shown.

It is interesting to compare the standardised indices for the line-transect aerial survey [17] and the commercial spotting data. Although they do not always show identical patterns on a year by year timescale, they have broadly similar patterns over the whole period for which they overlap (Fig. 5). On average, the CV of the commercial index is larger (around 35%) than that of the aerial survey (around 20%).

## Discussion

A comparison between the nominal and standardised indices (Fig. 4) shows the need for modelling of the raw data to remove effects of different spotters and spotting conditions in order to obtain a comparable time series of values. Wind speed, spotting conditions and temperature are the most important environmental covariates for the SBT dataset. In addition to checking model diagnostics, it is important to ask whether the estimated relationships between response and explanatory variables are plausible. The negative relationship between sightings and wind speed is consistent with common sense and with spotters’ experience and comments. Stronger winds lead to choppy waters making it harder to spot surface schools than in calm waters. Similarly, the positive relationship with spotting conditions (higher values meaning better spotting conditions) is as expected. Although spotting conditions and wind (speed) are correlated, models with only one of these covariates have worse residuals.

We used air temperature data in analyses presented here. High air temperature is often associated with no wind (or low wind speeds), and hence flat, calm conditions making it easier to see fish at the surface. SBT may also be at the surface on warm weather days, possibly because the very surface of the water also warm up, and they seek warm waters for thermoregulation [23]. Since wind speed is included in the model in relation to spotting conditions, it could be argued that instead of air temperature, sea surface temperature (SST) should be explored as an explanatory variable of fish surfacing behaviour. Analyses of electronic tag data from juvenile SBT in the GAB in summer, suggest that their distribution and possibly surfacing behaviour are likely to be related to SST. Basson et al. [20] identify a range of preferred temperatures with peak ‘preference’ at around 19°C. The estimated dome-shaped relationship in the commercial spotting data is consistent with what is observed in the electronic tag data though the absolute SST values are, of course, lower than the absolute air temperature values. We are currently refining the dataset to include estimates of sea surface temperature obtained from a CSIRO remote sensing 3-day composite product (http://imos.aodn.org.au/imos/) using the spatial dynamics ocean data explorer (SDODE) interface [24].

The inclusion or exclusion of target species as a factor has hardly any effect on the resulting index of abundance, but the model fit is somewhat improved and the AIC lower when it is included. This covariate has, in fact, been more important in the past when analyses were based on the subset of two spotters who had spotted in all seasons between 2003 and 2012. Flights targeting both skipjack and SBT are estimated to be slightly more effective (116%) than those just targeting SBT. This may be because SBT and skipjack tend to occur in the same areas within the GAB, although schools of SBT and skipjack are very rarely found together. In contrast, when both SBT and mackerel, or all three species are targeted, the spotting rate is only about half that of pure SBT-targeted flights. This may be because mackerel is generally found in different areas (i.e. to the west) of the main SBT fishing ground.

The need for including an interaction term between month and season became apparent in past analyses but could only be handled in a GLM or GAM framework by leaving out data for March in all seasons due to no effort data in the March 2010 and 2013 seasons. The GAMM approach makes it possible to include the interaction term without omitting data. The inclusion of the interaction terms improved the diagnostics sufficiently to warrant use of the more complicated model.

Finally, it is worth commenting on the way in which the raw SBT sightings and flight path data have been compiled for the analyses presented here. There are, in fact, many different ways in which these data could be compiled for analysis and the construction of a relative index of juvenile abundance in the GAB. Most importantly, sightings need to be related to a measure of “search effort”. The ideal approach would be to compile the data at as fine a time and spatial scale as possible. This would enable us to adjust for the lack of full spatial coverage of flights (see Fig. 1) and to address the autocorrelation in the sightings. This task would, however, be highly complex and time-consuming, and not warranted (or affordable) given that a scientifically designed line-transect aerial survey has been conducted concurrently and provides the main, preferred, index of juvenile abundance for use in the SBT operating model (OM) and management procedure (MP) [6], [17]. The advantage of compiling the data by day over the whole area is that it allows for fast, relatively simple – and therefore not unduly costly – analysis.

## Conclusions

We have shown how a general additive model with random effects (GAMM) can be used to model an unbalanced dataset of sightings of SBT surface schools from the air. This approach allows for the inclusion of interaction terms and data from all spotters in the model without the need to remove ‘strata’ with missing data (as would be necessary in a GLM or GAM framework). The Tweedie distribution elegantly handles flights where no biomass was observed, i.e. zeros in the dataset. The implementation of random effects in the GAM model was relatively easy with the “mgcv” package in R, and this approach is equally applicable to the standardisation of more traditional fisheries catch per unit effort data, particularly in the case of unbalanced datasets.

The GAMM model was used to obtain a standardised, fisheries *dependent*, index of relative abundance for juvenile southern bluefin tuna in the Great Australian Bight in summer. It has been a useful adjunct to the aerial line-transect survey, which is, by its nature of a consistent design and logistics as well as explicit spatial structure, the preferred index of recruitment for assessment and management purposes. The index based on the data from commercial spotting, which is part of the catching operations, is subject to many of the problems commercial catch per unit effort data are subject to, such as changes in: spatial and/or temporal coverage, spotters, operational considerations and the needs of the catching operations. Nonetheless, we consider that the commercial spotting index has been useful over the past decade. Fishing operators sometimes question whether designed surveys (not just aerial surveys, but also trawl surveys) are representative, commenting that ‘the aircraft (or vessel) didn't go where the fish were’ on a particular day. The logic of a transect survey is of course exactly that; i.e. not to go searching for fish, but to “sample at random” in the same way each year. The commercial spotting index has shown very similar time trends to the line-transect survey index for the period over which they overlap (Fig. 5; [17]). Thus, those who questioned whether the aerial survey was representative would have been encouraged by the similarity in broad trends. In our view, this increased confidence in the line-transect aerial survey has meant stronger support for its inclusion in the operating model used by the Commission for the Conservation of Southern Bluefin Tuna (CCSBT) for assessment and simulation-testing purposes, and in the decision rule used to set global quotas [25]. There is no guarantee that the commercial index will always track the aerial survey index. This, together with the data issues and larger CV of the commercial index means that it would be unwise to consider replacing the fishery independent aerial line-transect survey with the commercial spotting index.

Although the most recent estimates of relative SSB are still low (between 3.5 and 7.7%; [25]), the important difference is that the CCSBT has now adopted a simulation tested management procedure that should, if adhered to, achieve the Commission's rebuilding target of a 70% probability of SSB being at 20% of the unfished level by 2035 [25]. In the context of rebuilding the SSB, continued monitoring of incoming recruitment remains important. We argue that the collection and analyses of data from commercial operations has played a positive role in this process.
